# Assessment of adolescents’ communication on sexual and reproductive health matters with parents and associated factors among secondary and preparatory schools’ students in Debremarkos town, North West Ethiopia

**DOI:** 10.1186/1742-4755-11-2

**Published:** 2014-01-08

**Authors:** Kasiye Shiferaw, Frehiwot Getahun, Getahun Asres

**Affiliations:** 1 Department of Midwifery, College of Health and Medical Sciences, Haramaya University, Haramaya, Ethiopia; 2 Department of Nursing, College of Medical and Health Sciences, University of Gondar, Gondar, Ethiopia; 3 Department of Epidemiology and Biostatistics, Institute of Public Health, University of Gondar, Gondar, Ethiopia

**Keywords:** Communications, Sexual and reproductive health issues, Associated factors, Northwest Ethiopia

## Abstract

**Background:**

Sexuality and reproductive health are among the most fundamental aspects of life. Poor parental involvement in preparing young people for safe sexual life and good reproductive health was part of the blame for the lack of skills on sexual decision making. Despite the growing needs, there is no adequate health service or counseling specifically suitable for this specific age group and research on the role of parents in this process has yielded inconsistent results.

**Objective:**

The objective of the study is to assess adolescents’ communication on sexual and reproductive health issues with parents and associated factors among secondary and preparatory schools students in Debremarkos town.

**Methods:**

School based study was conducted among secondary and preparatory schools students in Debremarkos town, from April 8 to 21, 2012. Multistage sampling and self administered questionnaires were employed.

**Results:**

The proportion of the students who had discussion on sexual & reproductive health issues with their parent was found to be 254 (36.9%). Mother who able to read and write (AOR = 2; 95% CI 1.3 to 3.1), adolescents accepting discussion of sexual & reproductive health issues (AOR = 2.5 95% CI 1.3 to 4.5), adolescents who ever got SRH information (AOR = 2; 95% CI 1.4 to 2.9), adolescents who ever had sexual intercourse (AOR = 1.7; 95% CI 1.1 to 2.6) were found to have significant positive associations, and being grade 12 students (AOR = 0.4; 95% CI 0.2 to 0.7) and having less than three family size (AOR = 0.5; 95% CI 0.2 to 0.9) showed significant negative associations.

**Conclusion and recommendation:**

Study unveils that parent –adolescent communications on sexual and reproductive health issues is low, only about one third of the students were communicating on SRH issues. Therefore; there is a need to equip and educate parents on different sexual & reproductive health issues. Comprehensive family life education should also be initiated for the students and parents.

## Introduction

Sexuality and reproductive health are among the most fundamental aspects of life. Sexual Health is about the enhancement of life and personal relations, and not merely counseling and care related to reproduction and STDs. Young people aged 10–24 years constitute around 1.8 billion and represent 27 percent of the world’s population. Studies suggested that adolescents have limited knowledge about SRH and know little about the natural process of puberty. This lack of knowledge about reproductive health may have grave consequences [1-3].

Adolescents, having survived all childhood health problems, have been enjoying a relatively low morbidity and mortality period in the past. At present, as a result of civilization, urbanization and change in life style, the health of adolescents is increasingly at stake. Sexually transmitted diseases, HIV/AIDS and other reproductive health problems are the greatest threats to their well-being [4].

Globally, rates of sexually transmitted infections among young people are soaring: one-third of the 340 million new STIs each year occur in people under 25 years of age. Each year, more than one in every 20 adolescents contracts a curable STI. More than half of all new human immunodeficiency virus (HIV) infections occur in people between the ages of 15 and 24 years. While urbanization brings greater access to education and health services, it also carries greater exposure to the risks of drug and alcohol abuse, violence, and STIs, including HIV/AIDS. Modernization tends to create more employment opportunities, but it may also bring about a loss of traditional cultures and separation from extended families [5,6].

In Sub Saharan Africa many young adolescents do not know how to protect themselves and their partners against HIV/AIDS and other STDs. For instance, despite that AIDS awareness is relatively high among youth in Ethiopia, one in four young women and more than one in ten young men have not heard of AIDS or know whether AIDS can be avoided. While 45% of young women are aware that using a condom in every intercourse prevents HIV, only 2 percent of them report having used condom at last intercourse. Unlike most other illnesses and disabilities, sexual and reproductive health problems tend to be cloaked in embarrassment, secrecy, and shame. Because of cultural taboos adolescents in many developing countries rarely discuss sexual matters explicitly with their parents. Most information for their patchy knowledge comes from peers of the same sex who may themselves lack adequate information or are incorrectly informed. Increased sexual activity places youth at greater risk of unintended pregnancies and STIs, including HIV/AIDS. Many unintended pregnancies end in abortion, and unsafe abortions, which are sometimes self-induced, can result in severe illness, infertility, and death. Many of these problems can be addressed, however, through sound evidence and open dialogue [1,3,6-8].

In Ethiopia childbearing begins at an early age, 45% of the total births in the country occur among adolescent girls and young women, 60% of adolescent pregnancies are unwanted or unintended and participants who didn’t found easy to discuss about important matters with their parent were more likely to initiate sex-earlier. Addressing immediate and long-term RH needs of young people & strengthening multisectoral partnerships to respond to young women’s heightened vulnerability to sexual violence and non-consensual sex are some of strategies in Ethiopia. Some of the activities in Ethiopia are creating awareness of RH, providing youth-friendly services, increasing human resource capacity, explore new opportunities, & expand multisectoral coordination [4,8,9].

Poor parental involvement in preparing young people for safe sexual life and good reproductive health was part of the blame for the lack of skills on sexual decision making. Life skills enable individuals to translate knowledge, attitudes and values in to actual abilities i.e. “what to do and how to do it”. Life skills are abilities that enable individuals to behave in healthy ways, given the desire to do so and given the scope and opportunity to do so. There are taboos of purposeful teen-parent communication on sexual matters including condom use at home. Their only general and superficial information came from mass media, peers and few anti-AIDS clubs teaching in the schools. Parents and other family members are in a unique position to help socialize adolescents into healthy sexual adults, both by providing accurate information about sex and by fostering responsible sexual decision making skills [10-12].

However, despite the growing needs, there is no adequate health service or counseling specifically suitable for this specific age group unlike children, mothers or adults, and research on the role of parents in this process has yielded inconsistent results. A number of NGO and government work to integrate reproductive health care information and services into the various other services provided at the service-delivery level. However; most participants believed that service providers at health posts do not keep information confidential and do not behave nicely if sexual health problems are shared with them [4,13,14].

So, this study is aiming at assessing adolescents’ communication on SRH issues with parents and associated factors that may help policy makers, program planners and implementers to design appropriate interventions to address the SRH issues of adolescents.

## Methods

### Study design

School based study was conducted among secondary and preparatory schools students in Debremarkos town, East Gojam zone, Amhara National Regional State from April 8 to 21, 2012.

### Study area and period

The study was conducted in Debremarkos town, East Gojam zone, Amhara National Regional State from April 8 to 21, 2012. Dabremarkos is capital town of East Gojam, and located 300 km North West of Addis Ababa. In the town there are two high schools and one preparatory school which are government schools. The total numbers of students attending in all high schools and preparatory school in Debremarkos town were 6001. According to the information obtained from the town statistics office report, the current (2007) total population of the town was 62,469 with sex distribution of 47.87% “males” and 52.13% “females”. There are one hospital and three health center in the town. There are also four NGO clinics and one FGAE. None of these institutions were practicing youth friendly service practically.

### Study population

The study population was all students from grade 9 to grade 12 attending secondary and preparatory schools in Debremarkos town during the study period.

#### ***Inclusion criteria***

Unmarried adolescents in the age group 10–24 years were included.

#### ***Exclusion criteria***

Sick and involuntariness were excluded during data collection.

### ***Sample size and sampling procedures***

#### Sample size determination

The sample size was determined using single population proportion formula considering the following assumptions: P = 29% (proportion of students communicating on SRH issues with parents which is taken from previously done study [15])

Significance level 5% (α = 0.05), and Z α*/2* = 1.96

Margin of error 5% (D = 0.05).

The formula for calculating the sample size where N <10,000 (N =6001) is:

$$\begin{array}{c} n=\frac{{\left(z\alpha /2\right)}^{2}P\left(1‒P\right)}{d^{2}}\\ =\frac{1.96^{2}*0.29*0.71}{{\left(0.05\right)}^{2}}=317,\;n=317 \end{array}$$

Assuming 10% non-response rate, design effect 2, the sample size was:

$$n=317^{*}2+10\%=634+63=697$$

The final sample size was 697 secondary and preparatory schools students in Debremarkos town.

#### ***Sampling procedures***

A multistage sampling was used. Simple random sampling was used to select the sections from each grade in the three schools. Finally, the study subjects were selected using simple random sampling technique.

### Variables of the study

#### ***Dependent variable***

Communication on SRH issues

#### ***Independent (explanatory) variables***

● Socio-demographic variables: Age, sex, religion, ethnicity, residence

● Family factors

– educational status of father

– educational status of mother

– occupation of father

– occupation of mother

– family income

– family size

● Living arrangements of the students

● Knowledgeable on SRH issues

● Grade of students

● Ever had SRH information

● Ever had sexual intercourse

### Operational definition

#### ***Communication on SRH issues***

Students who discussed at least two SRH issues (condom, STI/HIV/AIDS, abstinence, unwanted pregnancy, contraception) with their parents in the last 12 months

### Parents

Parents in this study mean biological parents, step parents or foster parents but it does not include elder siblings

### Knowledgeable on SRH

Those students who score above the mean of the sexual and reproductive health related questions.

### Data collection procedures

#### ***Data collection instrument***

A structured and self administered questionnaire with closed-ended questions was used to collect the data from the respondents after pretest. The questionnaire was prepared in English and it was translated to Amharic language for appropriateness and easiness. The Amharic version was again translated back to English to check for consistency of meaning. Translation of questionnaire was done by language experts in both cases. The questions included in the questionnaire were prepared depending on review of different related literatures and variables identified to be measured.

#### ***Data collectors***

Four individuals, who had completed grade ten, were selected for data collections, preferably who has experience of data collection. Confidentiality and privacy was given attention during training and the trainees were participated on pre-testing of the questionnaire. Data collectors approached the selected respondents by explaining the aims of the study and what sort of information is needed from them. Data collectors were supervised by two diploma midwives and problems faced during data collection were solved on time. The principal investigator was checking filled questionnaires and solution was given by discussing with the supervisors and the data collector if problem arise. Finally, filled questionnaires were signed by supervisors after checking for its completeness.

#### ***Data quality control issues***

To ensure the quality of data, two days training was given to supervisors and the data collectors on how to approach the study subjects, on the objective of the study, the content of the questionnaire, selection of the study subjects and on issues related to communication on SRH. The investigator also discussed with the supervisors as to how supervise the data collectors to ensure that the collectors are on duty and to solve problems faced during the data collection process. Structured and self-administered questionnaire was employed. Data collectors were supervised by two diploma midwives and problems faced during data collection were solved on time. Moreover, a pretest of questionnaire conducted on seventy students from Debremarkos zuria district secondary school to ensure its completeness and consistency in providing the information needed for the study.

### Data processing and analysis

The data, which is gathered using structured questionnaire was coded and entered to Epi-Info version 3.5.1 and transferred to SPSS version 16, and checked & cleaned for its completeness and errors in coding and entering before analysis. The study population was explained in relation to relevant variables, frequencies tables, graphs and summery statistics.

Most of the variables were fitted to the bivariate logistic regression. Then all variables having p value ≤ 0.2 in the bivariate analysis were further entered into multivariate logistic regression model. In the multivariate analysis, standard enter techniques were fitted. Variables having p value ≤ 0.05 in the multivariate analysis were taken as significant predictors. Crude and adjusted odds ratios with their 95% confidence intervals were calculated. The Hosmer and Lemeshow goodness-of-fit test was used to assess whether the necessary assumptions for the application of multiple logistic regression were fulfilled and p value > 0.05 was considered a good fit.

### Ethical consideration

Ethical clearance was obtained from Institutional Review Board of University of Gondar. Official letter that explains the objectives, rationale and expected outcomes of the study was written to zonal education office from the college of medicine and health science, University of Gondar, which requests cooperation. The principal investigators communicated zonal education office and obtained written consent from the office as well as from secondary and preparatory schools on which the study was conducted.

The data collectors approached the selected respondents first by greeting and continue on explanation of the aims of the study and what is needed from them. Information was collected after obtaining verbal consent from each participant to be included. Respondents were also being informed that they can refuse or discontinue participation at any time they want. Information was recorded anonymously and confidentiality was assured throughout the study period.

## Results

### Socio-demographic variables

The response rate of the study was (98.99%). Of these 343 (49.9%) were males. The mean age of the respondents was 17.29 ± 1.59 SD. Six hundred eighty one (99%) of the respondents were Amhara Ethnic group and 676 (98.3%) were Orthodox Christians. Respondents who were living with their father and mother account 404 (58.7%) followed by those who live with their mother 103 (15%). Two hundred forty five (35.6%) were grade ten students followed by grade nine 208 (30.2%). About 545 (79.2%) of the respondents were urban resident (Table 1).

**Table 1:** Socio-demographic characteristics of secondary and preparatory schools’ students in Debremarkos Town, July, 2012

**Variable**	**Number (n=688)**	**Percent**
**Sex**		
Male	343	49.9%
Female	345	50.1%
**Age**		
10-14	10	1.4%
15-19	621	90.3%
20-24	57	8.3%
**Grades**		
Grade 9	208	30.2%
Grade 10	245	35.6%
Grade 11	119	17.3%
Grade 12	116	16.9%
**Religions**		
Orthodox Christian	676	98,3%
Others	12	1.6%
**Ethnic groups**		
Amhara	681	99%
Others	7	0.9%
**Residence**		
Urban	545	79.2%
Rural	143	20.8%
**Living arrangement of respondents**		
With both parents	404	58.7%
With father	20	2.9%
With mother	103	15%
With friend/s	21	3.1%
Alone	84	12.2%
With relatives	44	6.4%
Others	12	1.7%
**Family size**		
Less than three	73	10.6%
Four to six	458	66.6%
Greater than seven	157	22.8%
**Family income**		
Less than 500	28	19.3%
501-1000	39	26.9%
>1001	78	53.8%
**Educational status of father**		
Unable to read and write	101	14.7%
Able to read and write	225	32.7%
Grade 1-6	87	12.6%
Grade 7-12	79	11.5%
Above grade 12	196	28.5%
**Educational status of mother**		
Unable to read and write	203	29.5%
Able to read and write	188	27.3%
Grade 1-6	83	12.1%
Grade 7-12	101	14.7%
Above grade 12	113	16.4%
**Occupation of father**		
Employed government	189	27.5%
Employed private	82	11.9%
Merchant	122	17.7%
Farmer	245	35.6%
Others	50	7.3%
**Occupation of mother**		
House wife	347	50.4%
Employed government	83	12.1%
Employed private	43	6.2%
Merchant	75	10.9%
Farmer	124	18%
Others	16	2.3%

The mean family size of respondents was 5.5 ± 2.15 SD. Only, 145 (21.1%) of the respondents knew their family monthly income, and the mean family monthly income was 1964.90 Birr. One hundred ninety six (28.5%) and 113 (16.4%) of respondents’ father and mother were above grade 12 respectively. On the other hand, 245 (35.6%) of the respondents’ father were farmer, whereas, 347 (50.4%) of the respondents’ mother were housewife (Table 1).

### Knowledge of respondents on SRH issues

Four hundred twelve (59.9%) knew when first menstrual period started (menarche), and the reported mean age of menarche was 13.48 ± 1.69 SD. Around 24.6% and 35.3% of males and females respectively knew when first menstrual period started, but 25.3% of males and 14.8% females don’t know age of menarche. Feeling towards first menarche among female respondents include stressed, fear, felt sick and ashamed which account 146 (21.2%), 106 (15.4%), 70 (10.2%), and 67 (9.7%) respectively.

Three hundred ninety four (57.3%) of the respondents were knowledgeable about SRH issues. Moreover; six hundred seventy three (97.8%) have heard STI/HIV/AIDS. Majority of the respondents heard about HIV/AIDS account 664 (96.5%) followed by syphilis account 356 (51.7%). But, only 240 (34.9%) of the respondents have heard of LGV.

Majority of the respondents, 572 (83.1%) have heard of STI symptoms and mostly heard STI symptoms were burning sensation during urinations account 433 (62.9%) followed by genital ulcer account 399 (58%). Six hundred fifty six (95.3%) of the respondents have heard of HIV/AIDS prevention methods. The mostly heard prevention methods were being faithful to partner, abstinence, avoiding of sharing sharp materials which accounts 543 (78.9%), 541 (78.6%) and 482 (70.1%) respectively. Those who heard of consistent use of condom account 449 (65.3%).

On the other hand six hundred thirty one (91.7%) of the respondents have heard of contraceptive methods and norplant was the commonly heard method of contraceptive followed by injectables which account 507 (73.7%) and 497 (72.2%) respectively. And those who heard of emergency contraceptives were 342 (49.7%).

### Information source of SRH issues

**Figure 1:**
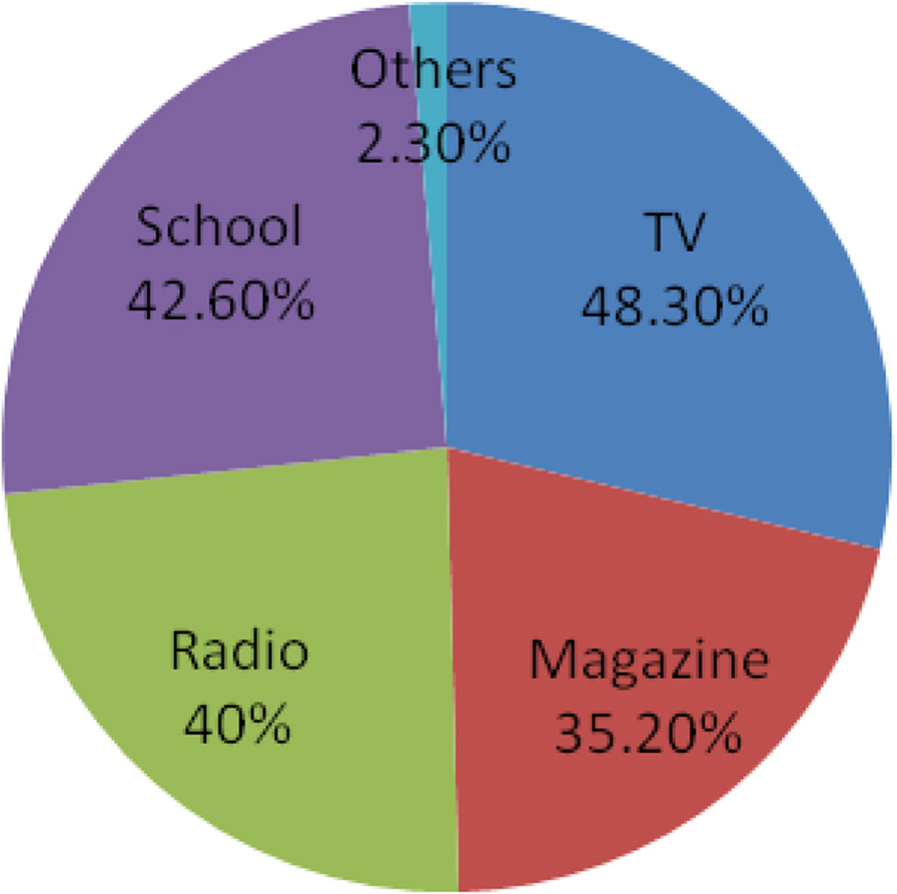
Source of SRH information for secondary and preparatory schools’ students (n = 688) in Debremarkos town, 2012.

Four hundred twenty six (61.9%) of the respondents mentioned that they have got sexual and reproductive health information. Among mentioned source of information for SRH issues, TV and school account 332(48.3%) and 293 (42.6%) respectively (Figure 1).

**Figure 2:**
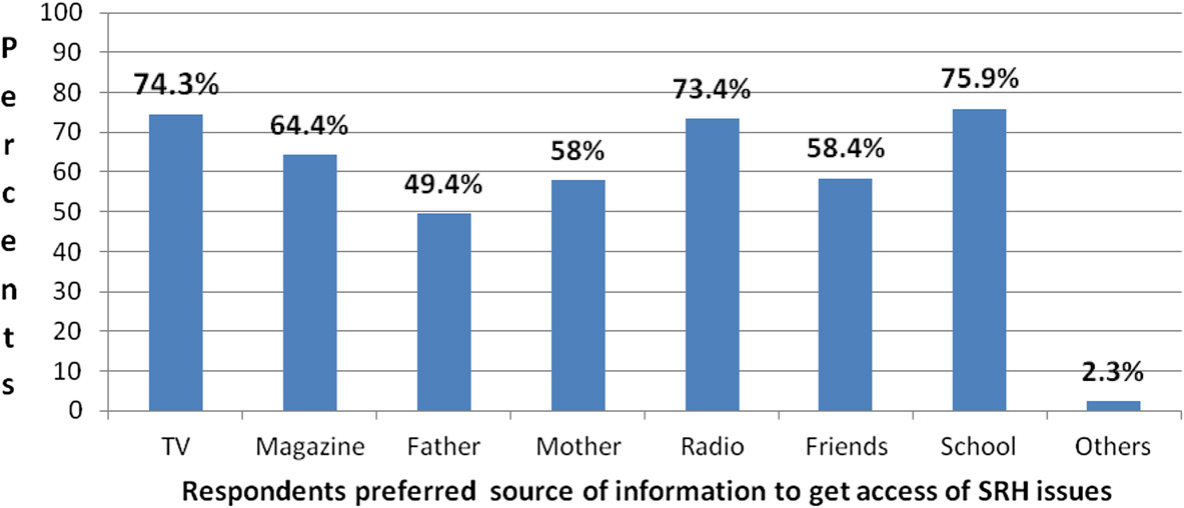
Respondents’ preferred source of information to get access of SRH issues in Debremarkos secondary and preparatory schools (n=688), July 2012.

However; only 28.8% and 24.3% of the respondents witnessed friends and mothers were their SRH issues information sources respectively. Majority of the respondents preferred to get access of SRH information from school account 522 (75.9%) followed by Television and Radio which account 511 (74.3%) and 505 (73.4%) respectively (Figure 2).

### Communications on SRH issues

Majority of the respondents 610 (88.7%) accepted that it is important to discuss SRH issues with the parents. However; only 254 (36.9%) of the students had discussion with either of the parents on at least two topics of SRH issues.

One hundred seventy eight (25.9%) of the students held discussion with their parents on contraceptives. Among those 141 (20.5%) of the students had discussion with their friend/s followed by mother 127 (18.5%). On the other hand, the respondents estimated reason for not discussing contraceptive with their parents were parents lack of knowledge which account 150 (21.8%) followed by parents lack of communication skills account 143 (20.8%) (Table 2).

**Table 2:** Secondary and preparatory schools students and with whom they had discussion in different topics of SRH in Debremarkos town, July, 2012

**Topic of discussion**	**Discussed**	**With whom they had discussed**					
	**Yes**	Father*	Mother *	Friend*	Sister*	Brother*	Teacher*
Contraceptive ^+^	178(25.9)	77(11.2)	127(18.5)	141(20.5)	95(13.8)	62(9)	45(6.5)
STI/HIV/AIDS^+^	238(34.6)	105(15.3)	145(21.1)	177(25.7)	126(18.3)	107(15.6)	66(9.6)
Unwanted Pregnancy^+^	196(28.5)	76(11)	140(20.3)	141(20.5)	94(13.7)	66(9.6)	36(5.2)
Premarital sex^+^	190(27.6)	78(11.3)	105(15.3)	145(21.1)	98(14.2)	75(10.9)	39(5.7)
Condom^+^	141(20.5)	49(7.1)	76(11)	131(19)	67(9.7)	61(8.9)	40(5.8)

*Multiple responses were possible.

+Percents are in the bracket.

The respondents who had had discussion on STI/HIV/AIDS were 238 (34.6%). Among those students, 177 (25.7%) had discussion with their friends, followed by mother account 145 (21.1%). Majority of the respondents 450 (65.4%) had no discussion about STI/HIV/AIDS, because of parents lack knowledge on SRH issues account 129 (18.8%) followed by parents lack communication skills account 121 (17.6%).

One hundred ninety six (28.5%) reported to have had discussion about unwanted pregnancy. Among those respondents, 141 (20.5%) had discussed unwanted pregnancy with their friends and 140 (20.3%) with their mother. One hundred fifty five (22.7%) didn’t know the reasons why they didn’t discuss unwanted pregnancy with their parents and 129 (18.8%) of the respondents reasoned out, parents lack of knowledge and communication skills (Table 2).

Among 190 (27.6%) of the respondents who had discussion on premarital sex, 145 (21.1%) and 105 (15.3%) had held discussion with friend/s and mother respectively. About 498 (72.4%) of the respondents didn’t discuss premarital sex, among those 155 (22.5%) of the respondents don’t know the reason why they didn’t discuss and 142 (20.6%) of the respondents said it is shameful to discuss (Table 3).

**Table 3:** Major reasons of respondents for not discussing SRH issues with their parents, in Debremarkos town secondary and preparatory schools, July 2012

		**Respondents’ reasons for not discussing sexual and reproductive health issues with their parents**						
**SRH issue**	**Not discussed**	*Culturally not acceptable	*Shame	*Parents lack of knowledge	*Parents lack of communication skills	*Parents are not good listener	*Parents are busy	*Don’t know
Family planning^+^	510(74.1)	64(9.3)	139(20.2)	150(21.8)	143(20.8)	54(7.8)	66(9.6)	108(15.7)
STI/HIV/AIDS^+^	450(65.4)	49(7.1)	100(14.5)	129(18.8)	121(17.6)	58(8.4)	58(8.4)	116(16.9)
Unwanted pregnancy^+^	492(71.5)	55(8)	112(16.3)	129(18.8)	129(18.8)	55(8)	51(7.4)	155(22.7)
Premarital sex^+^	498(72.4)	56(8.1)	142(20.6)	105(15.3)	110(16)	62(9)	46(6.7)	155(22.5)
Condom^+^	547(79.5)	63(9.2)	162(23.5)	122(17.7)	118(17.2)	64(9.3)	46(6.7)	167(24.3)

*Multiple responses were possible.

+Percents are in the bracket.

On the other hand, one hundred forty one (20.5%) of the participants claimed to have had discussion on condoms. One hundred thirty one (19%) discussed with their friends, followed by mother and sisters which account 76 (11%) and 67 (9.7%) respectively. Most of the respondents had no discussion on condom, the reasons they mentioned were unknown which account 167 (24.3%) and shame to discuss account 162 (23.5%).

Regarding the preferred group for discussion about SRH issues, 391 (56.8%) of the participants chose their friends followed by sisters which account 161 (23.4%). Three hundred one (43.8%) accepted that mothers were open to discuss SRH issues when compared to fathers account 219 (31.8%). On the other hand 357 (51.9%) of respondents rate their parent communications skill on SRH issues as low whereas, 256 (37.2%) and 75 (10.9%) students said medium and high respectively. Moreover; 274 (39.8%) of respondents had mentioned that their parents were not allow them to play with opposite sex. The mean age of which respondents recommend to start SRH issues communication with parents was 13.65 ± 4.42 SD.

### Sexual behavior

Two hundred sixty two (38.1%) students believed that it is normal and acceptable to have sexual feeling during adolescence. Majority of the respondents 607 (88.2%) believe sexual intercourse should be delayed till marriage. However; 118 (17.2%) of the respondents had premarital sex. Engaging in sexual activity was reported to be 87 (25.4%) among males compared to females 31 (9%). The mean age of sexual commencement was 15.2 ± 3 SD and the median was 16 years old.

Among those who were sexually active 96 (81.35%) had sexual intercourse in the past 12 months and those who had sex with one partner, two partner and three partner and more account 49 (51.1%), 23 (23.6%) and 24 (25%) respectively. Fifty eight (60.42%) of those who had sexual intercourse in the past 12 months had made ever use of condom and 54 (56.25%) used condom consistently.

### Factors associated with communications

In the bivariate analysis school grade of respondents, with whom respondents live, family size, educational status of mother, adolescents’ perceived importance to discuss SRH issues with parents, adolescents’ ever having sexual intercourse and adolescents’ ever getting SRH information were found to be significantly associated with communication on SRH issues (Table 4).

**Table 4:** Bivariate and multivariate analysis of factors associated with communication on SRH issues with parents among secondary and preparatory schools students in Debremarkos town, July 2012

**Variable**		**Communication on SRH issues with parents**	
		**Crude OR(95% CI)**	**Adjusted OR (95% CI)**
**Age**	10-14 years	2.0(0.5, 7.7)	1.1(0.2, 5.0)
	15-19 years	1.1(0.6, 2.0)	0.8(0.4, 1.5)
	20-24 years	1	1
**Sex**	Male	1	1
	Female	1.2(0.9, 1.7)	1.2(0.9, 1.7)
**Grade**	9^th^	1	1
	10^th^	0.8(0.5, 1.2)	0.7(0.5, 1.1)
	11^th^	0.7(0.4, 1.1)	0.6(0.4, 1.0)
	12^th^	0.4(0.2, 0.7)^**^	0.4(0.2, 0.7)^**^
**With whom respondents live**	Father & mother	1.3(1.0, 1.9)^**^	1.3(0.9, 1.8)
	Others	1	1
**Family size**	< 3	0.6(0.3, 1.2)	0.5(0.2, 0.9)^**^
	4-6	1.1(0.7, 1.6)	1.0(0.6, 1.5)
	>7	1	1
**Residence**	Urban	1.1(0.7, 1.7)	1.0(0.6, 1.6)
	Rural	1	1
**Educational status of father**	Unable to read and write	1	1
	Able to read and write only	0.9(0.5, 1.6)	0.8(0.4, 1.4)
	Grade 1-6	1.7(0.9, 3.1)	1.4(0.7, 2.9)
	Grade 7-12	1.3(0.7, 2.4)	1.0(0.4, 2.2)
	Above grade 12	1.1(0.6, 1.8)	0.7(0.3, 1.6)
**Mother educational status**	Unable to read and write	1	1
	Able to read and write	2.2(1.4, 3.3)^**^	2(1.3, 3.1)^**^
	Grade 1-6	1.3(0.7, 2.3)	1.1(0.6, 1.9)
	Grade 7-12	1.7(1.0, 2.8)^**^	1.4(0.8, 2.3)
	Above 12	1.6(1.0, 2.7)^**^	1.3(0.8, 2.2)
**Occupation of father**	Employed (private)	1.1(0.5, 2.1)	1.1(0.5, 2.5)
	Employed (government)	0.7(0.3, 1.5)	0.8(0.3, 1.8)
	Small scale merchant	1.0(0.5, 2.0)	1.0(0.4, 2.2)
	Farmer	0.8(0.4, 1.6)	0.9(0.4, 2.1)
	Others	1	1
**Occupation of mother**	Housewife	1	1
	Employed (private)	1.3(0.8, 2.1)	1.4(0.7, 2.8)
	Employed (government)	1.1(0.5, 2.1)	1.2(0.6, 2.5)
	Small scale merchant	0.8(0.5, 1.4)	0.7(0.4, 1.4)
	Farmer	0.9(0.6, 1.4)	1.0(0.6, 1.7)
	Others	1.3(0.4, 3.7)	1.3(0.4, 4.1)
**Knowledgeable about SRH issues**	Yes	0.9(0.7, 1.3)	1.0(0.7, 1.5)
	No	1	1
**Importance to discuss SRH issues with parent**	Yes	2.7(1.5,4.8)^**^	2.5(1.3, 4.5)^**^
	No	1	1
**Had ever got SRH information**	Yes	2.2(1.6,3.1)^**^	2(1.4, 2.9)^**^
	No	1	1
**Ever had sexual intercourse**	Yes	1.5(1.0, 2.2)^**^	1.7(1.1, 2.6)^**^
	No	1	1

**significant association.

The result of multiple logistic regression models revealed that being grade 12 students, having family size less than three, mother who was able to read and write, adolescents’ perceived importance to discuss SRH issues with parents, adolescents’ ever having sexual intercourse and adolescents’ ever getting SRH information were variables significantly associated with communication on SRH issues with parents (Table 4).

Grade 12 students were less likely to communicate on SRH issues compared to grade 9 students (Adjusted OR = 0.4; 95% CI 0.2 to 0.7). Those students whose family size was less than three were less likely to communicate compared to those whose family size was greater than seven (Adjusted OR = 0.5; 95% CI 0.2 to 0.9). Students, whose mother was able to read and write were 2 times more likely to communicate SRH issues with their parents than those students whose mother were unable to read and write (Adjusted OR = 2; 95% CI 1.3 to 3.1).

Students, who accepted the importance of discussing sexual and reproductive health issues with their parents, were 2.5 time more likely to discuss SRH issues than those who had not accepted the importance (Adjusted OR = 2.5 95% CI 1.3 to 4.5).

Those students who had ever got SRH information were 2 times more likely to communicate SRH issues with their parents than those who had never got SRH information (Adjusted OR = 2; 95% CI 1.4 to 2.9). The study also revealed that those students who have ever had sexual intercourse were 1.7 times more likely to communicate on SRH issues with their parents than those who have never had sexual intercourse (Adjusted OR = 1.7; 95% CI 1.1 to 2.6).

## Discussion

In this study, 36.9% of respondents had discussed SRH issues with their parents in the last 12 months. This finding is higher than a study conducted in Bullen wereda (29%), Hawasa (30.4%), Lesotho (20%), but, lower than study conducted in Zimbabwe (44%), Malawi (74%) [5,15-17]. This may be due to demographic and cultural difference and difference in accessing SRH information.

The result of multiple logistic regression models revealed that only, mother who was able to read and write, adolescents’ perceived importance of communication on SRH issues, adolescents’ ever having sexual intercourse and adolescents’ ever getting SRH information showed significant positive association with communication on SRH issues, but being grade twelve students, and having family size less than three showed significant negative associations.

Grade twelve students were less likely to communicate on SRH issues compared to grade nine students. This may be due to the parents thought that their children are knowledgeable about SRH issues and SRH issues are given in school and grade twelve spend most of their time reading compared to others since they have entrance exam at the end of the year. Those students whose family size was less than three were less likely to communicate compared to those whose family size was greater than seven. This may be due to as number of children increase parents are more concerned and have communication on SRH issues compared to small family size.

Those students whose mother was able to read and write were more likely to communicate SRH issues with their parents than those students whose mother was unable to read and write. It is in line with study from Hawasa, Ethiopia [5]. This may be due to the difference in knowledge on sexual and reproductive health issues, skill of communication, perceived importance of communication on SRH issues, and accessing information about sexual and reproductive health issues.

Students, who accepted the importance of discussing SRH issues with their parents, were more likely to discuss SRH issues than those who had not accepted the importance. This may be due to perceived importance of communications on SRH issues.

Those students who have ever got SRH information were more likely to communicate SRH issues with their parents than those who have never got SRH information. This may be due to respondents who have some awareness might be more eager to communicate SRH issues and the information they got may pave the way for initiation of communication.

The study revealed that those students who ever had sexual intercourse were more likely to communicate on SRH issues with their parents than those who never had had sexual intercourse. This may be due to fear of risks come as a result of sex & exploration habits of the students/adolescents.

Limitations: Since we asked the respondents’ discussion on SRH issues in the past twelve months, we might have introduced recall bias, as they might not remember what they had discussed in the last twelve months. This may under- or over-estimate communication of SRH issues. It is also difficult to establish temporal relationship as the study design was cross-sectional. Despite these limitations, the findings from this study will contribute to the understanding of the factors associated with communications of SRH issues in the study area.

## Conclusion

In this study parent –adolescent communications on sexual and reproductive health issues was found to be low. Adolescents whose mother able to read and write and adolescents who ever got SRH information communicate on SRH issues with parents than others. Moreover; adolescents who perceived important to discuss SRH issues with parents and adolescents who ever had sexual intercourse communicate SRH issues with parents compared to others. But, grade twelve students and parents whose family size is less than three were less likely to communicate SRH issues compared to others.

### Possible actions

Equip and educate parents on different sexual and reproductive health issues.

Initiate comprehensive family life education for the adolescents and parents.

Further qualitative study should be done on adolescents and parents communication.

## Abbreviations

AIDS: Acquired immuno deficiency syndrome; GCMHS: Gondar college of medicine and health sciences; HIV: Human immuno-deficiency virus; RH: Reproductive health; SRH: Sexual and reproductive health; STI: Sexually transmitted infection; STD: Sexually transmitted diseases; WHO: World health organization.

## Competing interests

The authors declare that they have no competing interests.

## Authors’ contributions

KS designed the study, performed the statistical analysis and drafted the manuscript. FG and GA participated in the study design, implementation of the study, and contributed to the draft manuscript. All authors contributed to the data analysis, read and approved the final manuscript.

## Authors’ information

KS (MSc. in Midwifery) - Lecturer at Haramaya university, College of Health and Medical Science, Department of Midwifery. FG (MPH. in reproductive health) - Lecturer at Gondar University, Department of Nursing. GA (MD, MPH in Epidemiology and Biostatistics) - Lecturer at Gondar University, Institute of Public Health, Department of Epidemiology and Biostatistics.
